# Separation of Scales in Transpiration Effects on Low Flows: A Spatial Analysis in the Hydrological Open Air Laboratory

**DOI:** 10.1029/2017WR022037

**Published:** 2018-09-10

**Authors:** B. Széles, M. Broer, J. Parajka, P. Hogan, A. Eder, P. Strauss, G. Blöschl

**Affiliations:** ^1^ Centre for Water Resource Systems Vienna University of Technology Vienna Austria; ^2^ Institute of Hydraulic Engineering and Water Resources Management Vienna University of Technology Vienna Austria; ^3^ Umweltbundesamt, Environment Agency Austria Vienna Austria; ^4^ Federal Agency of Water Management Institute for Land and Water Management Research Petzenkirchen Austria

**Keywords:** low flows, diurnal fluctuations, evapotranspiration

## Abstract

The objective of this study was to understand whether spatial differences in runoff generation mechanisms affect the magnitudes of diurnal streamflow fluctuations during low flow periods and which part of the catchment induces the diurnal streamflow signal. The spatiotemporal variability of the streamflow fluctuations observed at 12 locations in the 66‐ha Hydrological Open Air Laboratory experimental catchment in Austria was explained by differences in the vegetation cover and runoff generation mechanisms. Almost a quarter of the volume associated with diurnal streamflow fluctuations at the catchment outlet was explained by transpiration from vegetation along the tributaries; more than three quarters was due to transpiration by the riparian forest along the main stream. The lag times between radiative forcing and evapotranspiration estimated by a solar radiation‐driven model increased from 3 to 11 hr from spring to autumn. The recession time scales increased from 21 days in spring to 54 days in autumn. Observations and model simulations suggest that a separation of scales in transpiration effects on low flows exists both in time and space; that is, the diurnal streamflow fluctuations are induced by transpiration from the riparian vegetation, while most of the catchment evapotranspiration, such as evapotranspiration from the crop fields further away from the stream, do not influence the diurnal signal in streamflow.

## Key Points

- The amplitude of the diurnal streamflow fluctuations from wetlands was 1 magnitude larger than those from tile drains and springs
- Three quarters of the volume associated with diurnal streamflow fluctuations was due to riparian transpiration along the main stream
- Lag times between radiative forcing and ET increased from 3 to 11 hr from spring to autumn

## 1 Introduction

Evaporation and transpiration in midlatitude humid catchments affect streamflow at two main time scales. At the seasonal time scale, the energy input is at a maximum in summer; therefore, evapotranspiration is also at a maximum. This depletes soil moisture in summer below the annual mean, which affects runoff generation during storms. Soil moisture depletion also tends to reduce groundwater recharge and hence discharge to the streams. Streamflow during recession periods is the net result of the interplay of the hydraulic aquifer characteristics and evapotranspiration within the catchment. Several studies observed faster streamflow recessions in summer than during the rest of the year due to summer evapotranspiration (Federer, 1973; Shaw & Riha, 2012; Szilágyi et al., 2007). At the daily time scale, there are similar fluctuations in the energy input between day and night, leading to diurnal fluctuations in evaporation and transpiration, which, again, affect soil moisture and subsurface flow. In small headwater catchments these diurnal fluctuations of transpiration usually imprint a diurnal signal on the streamflow during low flow periods (Gribovszki et al., 2010).

Even though there are two distinct time scales of variability in evaporation and transpiration, diurnal, and seasonal, a relationship between the two time scales would be expected. Only a few studies examined the seasonality in the diurnal fluctuations and how the diurnal transpiration was related to the seasonal transpiration that determines the catchment water balance. Bond et al. (2002) and Wondzell et al. (2007, 2010) showed that the area contributing to streamflow fluctuations decreased as the catchment gradually dried out. This dynamic was explained by the weakening of the coupling between the vegetation and stream during summer as the groundwater levels dropped. The time lags between transpiration and streamflow also varies seasonally due to changes in the flow paths (Barnard et al., 2010; Cadol et al., 2012; Deutscher et al., 2016; Fonley et al., 2016; Graham et al., 2013; Gribovszki et al., 2008; Kirchner, 2009; Szeftel, 2010; Szilágyi et al., 2008; Wondzell et al., 2007, 2010; Yue et al., 2016).

One of the main questions to investigate diurnal streamflow fluctuations during low flows in the past was to determine where the streamflow fluctuations originate. Numerous studies have confirmed that these fluctuations were induced by transpiration from the riparian and near river vegetation. Experiments where the riparian forest was removed showed that the streamflow fluctuations stopped (e.g., Dunford & Fletcher, 1947; Lawrence, 1990; O'Loughlin et al., 1982); however, when the hillslope vegetation was removed instead, the fluctuations persisted but in a modified way (Bren, 1997). Another question was related to the main flow paths in the subsurface between the vegetation and the stream. The integrated effect of site conditions and the species assemblage may determine the sources of root water uptake (Snyder & Williams, 2000). Certain trees may extract water directly from the groundwater (Barbeta & Peñuelas, 2017; Gribovszki et al., 2010; Snyder & Williams, 2000), but the amplitude of the water table fluctuations depends on the distance from the stream and the vegetation type. For example, Reigner (1966) showed that the groundwater fluctuations were significant only up to a 2‐m distance from the stream. Irrigation experiments of Barnard et al. (2010) in Oregon suggested, however, that during higher soil moisture conditions after irrigation, hillslope vegetation located further from the stream could contribute to the diurnal streamflow fluctuations. Yue et al. (2016) observed diurnal groundwater table fluctuations along a part of a river that was covered by woody species and wet slough; however, the middle section of the river with shallower‐rooted grasses did not exhibit water table fluctuations. When the depth to the water table exceeded a certain threshold during a long recession period, the amplitudes of the diurnal water table fluctuations observed in an area covered by wet slough decreased.

There are no robust methods for measuring the evapotranspiration rates in riparian forests with mixed vegetation types (Drexler et al., 2004; Goodrich et al., 2000; Landon et al., 2009; Leenhouts et al., 2006; Loheide et al., 2005). For many of the narrow riparian corridors, the fetch requirement of the eddy covariance method often exceeds the width of the riparian forest (Goodrich et al., 2000). Upscaling sap flux measurements from tree to stand level can also be problematic (Cermák et al., 2004; Oishi et al., 2008; Schaeffer et al., 2000). The empirical approaches based on crop coefficients and vegetation index‐based crop coefficients (Glenn et al., 2011; Nagler et al., 2005) are not widely used for riparian ecosystems due to the heterogeneous species composition. Therefore, estimating the evapotranspiration rates for the riparian zone based on measurements of diurnal water level fluctuations can be a valuable alternative (e.g., Cernohous & Šach, 2008; Dvorakova et al., 2014; Gribovszki et al., 2008; Loheide et al., 2005; White, 1932; Wondzell et al., 2007, 2010). However, estimating riparian zone evapotranspiration in terms of volumes might be uncertain at some study sites where estimated evapotranspiration rates are only representative of a fraction of the riparian zone (Butler et al., 2007; Loheide et al., 2005; White, 1932).

Previous studies examined diurnal streamflow fluctuations and their temporal changes in single or nested catchments with spatially rather uniform runoff generation mechanisms (e.g., Bond et al., 2002; Szeftel, 2010). However, it is not clear how spatial differences in the runoff generation mechanisms affect the flow paths and total evapotranspiration. The main objective of our study therefore was to evaluate spatial and temporal patterns of the diurnal streamflow fluctuations in relation to the seasonal cycle of evapotranspiration in a small experimental catchment with different runoff generation mechanisms and mixed land cover types. The study was performed in the Hydrological Open Air Laboratory (HOAL), a 66‐ha Austrian experimental catchment, where both the main stream and the tributaries draining nearly all the surface contributing area to the stream are gauged, allowing the main stream and tributary contributions to be separated. The goal of our study was to determine where the diurnal low flow fluctuations originate, that is, how much the streamflow fluctuations observed at the tributaries with different characteristics and runoff generation mechanisms (such as wetlands, springs, and tile drainage systems) contribute to the streamflow fluctuations observed at the catchment outlet. Furthermore, we aimed to understand which part of the catchment induces the streamflow fluctuations on the diurnal time scale and which part influences the evapotranspiration on the seasonal time scale. In order to explore the causal link between the process drivers and the streamflow signal, we used a solar radiation‐driven model. The estimated model parameters assisted in generalizing the response time scales associated with the diurnal fluctuations and the seasonal recession of streamflow.

## 2 Study Area and Data

### 2.1 Study Area

**Figure 1:**
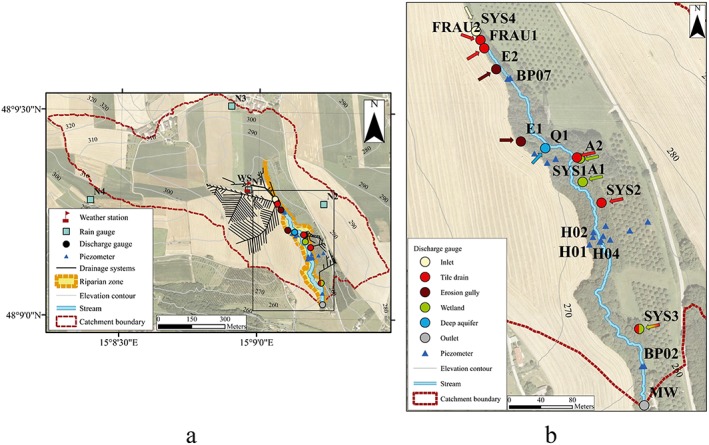
Hydrological Open Air Laboratory in Petzenkirchen, Lower Austria (panel a). Streamflow is monitored at the main outlet (MW) and at 11 tributaries (detail, panel b) that have different dominant runoff generation mechanisms.

The study was conducted in a small experimental catchment, the HOAL in Petzenkirchen, located in the western part of Lower Austria (Figure 1a), approximately 100 km west of Vienna (48°9′N, 15°9′E). The drainage area of the catchment is 66 ha at the catchment outlet (MW). The natural surface water outlet of the catchment is the Seitengraben stream. The elevation of the catchment ranges from 268 to 323 m above sea level, with a mean slope of 8%. The stream is approximately 620 m long and has a medium slope of 2.4% (Blöschl et al., 2016; Eder et al., 2014, 2010).

**Figure 2:**
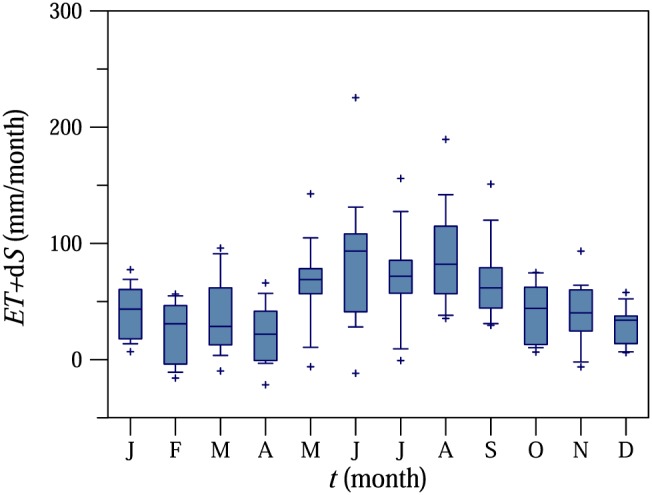
Monthly evapotranspiration (*ET*) and storage change (d*S*) estimated from the water balance in the Hydrological Open Air Laboratory for the period 2002–2015.

The climate is humid. Mean annual (2002–2015) air temperature, precipitation, runoff, and evapotranspiration are 9.6 °C, 784 mm/year, 178 mm/year, and 606 mm/year, respectively. Seasonal maxima of air temperature, rainfall amount, and intensity occur in the summer (Figure 3 in Blöschl et al., 2016). Mean monthly runoff tends to peak in winter or early spring. The seasonal variability in evapotranspiration plus storage change estimated from the water balance is presented in Figure 2.

The geology of the HOAL consists of Tertiary fine sediments and fractured siltstone of the Molasse zone. The dominant soil types are Cambisols (57%), Kolluvisol (16%), and Planosols (21%) with moderate to low permeability. Gleysols (6%) occur close to the stream (Blöschl et al., 2016; FAO et al., 1998).

The catchment is dominated by agricultural land use. At the time of the study 87% of the catchment area was arable land, the rest was forested, paved, or used as pasture. The crops were mainly winter wheat, maize, winter barley, and winter oilseed rape. Winter wheat and winter barley resume growing in March, reaching their full height in June and are harvested typically in July. Maize is planted middle/end of April and harvested in late September. Oil seed rape develops from March to June and is harvested in July. Most of the forested area is located close to the stream (Figure 1). The most common trees in the riparian zone are different species of willow (*Salix*), poplar (*Populus*), ash (*Fraxinus*), field maple (*Acer*), and black alder (Alnus glutinosa). The main shrub species in the riparian zone are cornel (*Cornus*), elder (*Sambucus*), hazelnut (Corylus avellana), honeysuckle (*Lonicera*), and guelder rose (*Viburnum*).

One of the main features of the HOAL is the wide range of observed runoff generation mechanisms, such as infiltration excess overland flow, reinfiltration of overland flow, saturation excess runoff from wetlands, tile drainage flow, shallow aquifer seepage flow, and groundwater discharge from springs (Blöschl et al., 2016). Due to the thin soil layers with low permeability and the agricultural land use, subsurface tile drainage systems were installed in the 1950s to eliminate waterlogging (Figure 1a). These systems contribute to the stream at five points: Sys1 and Sys2 flow throughout the whole year, while Frau1, Frau2, and Sys3 are ephemeral. Sys3 behaves as a combination of a tile drain and a wetland during low flow conditions as it also collects the water from the neighboring highly saturated parts of the catchment. Exner‐Kittridge et al. (2016) found that, chemically and dynamically, the water at Sys1 tile drain originates from the deep aquifer and not the shallow aquifer; therefore, Sys1 behaves like a spring rather than a drainage system. The upper 25% of the stream length was piped in the 1950s to expand the agricultural area. The concrete pipe enters the main stream at inlet Sys4. The dynamics of Sys4 are similar to the perennial drains. A perennial spring, Q1, originates from a fractured siltstone aquifer and directly enters the main stream. Two wetlands (A1 and A2) are fed by springs that seep into the stream through rivulets in the southeastern part of the catchment. Due to their high saturation, the runoff response of the wetlands is fast. During major storm events, saturation overland flow laterally enters the stream at E1 and E2 erosion gullies and, potentially, a few other points, depending on the event magnitude, soil moisture state, and the direction of the plowing. As both the main stream and the tributaries draining nearly all the surface contributing area to the stream are gauged (12 gauging stations in total), the main stream and tributary contributions can be separated.

The estimated drainage area, the percentage of the total drainage area occupied by the riparian forest, the forest cover, the mean streamflow, and the mean low flow episode streamflow (according to sections 3.1 and 4.1) are summarized in Table 1 for each gauge in the HOAL. Comparing the mean streamflow at MW catchment outlet with the sum of the tributaries for the time period 2013–2015, the tributaries contribute approximately 56% to the total runoff at MW outlet. The contribution of the tributaries to the main stream is higher in the low flow periods (63%). This means that about 40% of the streamflow observed at MW enters the stream laterally in a diffuse way, mostly through the subsurface.

**Table 1:** Estimated Drainage Area, Proportion of the Catchment Drainage Area Occupied by Riparian Forest, Forest Cover, Mean Streamflow and Mean Low Flow Episode Streamflow for 2013–2015 (for the MW Catchment Outlet Streamflow Data for 2002–2015 are in brackets) for Each Gauge in the HOAL (Figure 1 Shows the Location of the Gauges)

Gauge	Runoff generation mechanism	Estimated drainage area (ha)	Riparian zone (%)	Forest cover (ha)	Mean streamflow (L/s)	Mean low flow episode streamflow (L/s)
MW	Outlet	65.8	3.5	6.32	4.23 (3.94)	3.12 (2.82)
Sys4	Inlet pipe	37.4	0.2	1.73	0.74	0.62
Frau1	Tile drain	3.1	0.1	0.00	0.03	0.00
Frau2	Tile drain	4.8	0.3	0.01	0.15	0.09
Sys1	Tile drain (deep aquifer)	6.5	5.0	0.77	0.43	0.41
Sys2	Tile drain	2.4	0.7	0.45	0.18	0.16
Sys3	Tile drain/Wetland	4.3	0.1	0.61	0.09	0.06
A1	Wetland	2.1	1.6	0.25	0.09	0.09
A2	Wetland	1.1	7.0	0.17	0.09	0.06
Q1	Deep aquifer	2.0	0.8	0.02	0.46	0.46
E1	Erosion gully	0.8	0.9	0.01	0.01	0.00
E2	Erosion gully	1.0	0.2	0.00	0.09	0.00

### 2.2 Data

Streamflow has been monitored at MW outlet of the catchment by a calibrated H‐flume with a pressure transducer since 2001 with 1‐min temporal resolution (Figure 1). Streamflow measurements at the tributaries started in 2011. At Sys4 inlet, at Frau1, Frau2, and Sys2 tile drainage systems, Sys1 tile drain (deep aquifer), Sys3 tile drain/wetland, at the wetlands (A1 and A2) and at the erosion gullies (E1 and E2) H‐flumes and pressure transducers are used to monitor the flow; at Q1 spring a V‐notch weir and a pressure transducer are used to measure the flow. Details on the sensors are given in Blöschl et al. (2016).

**Figure 3:**
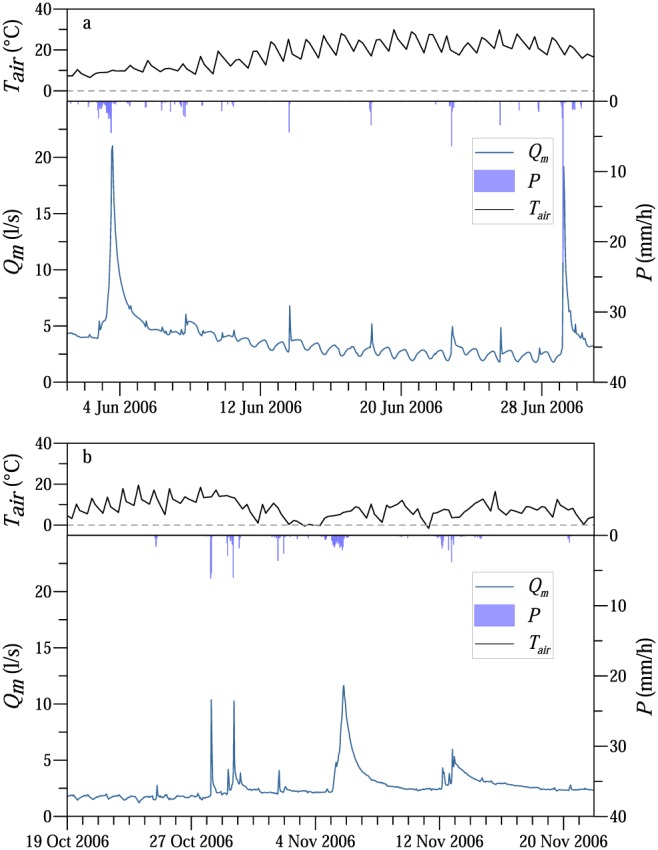
Example of air temperature (*T_air_*) interpolated between 7, 14, and 19 hr, rainfall (*P*), and streamflow (*Q*_*m*_) fluctuations at the main outlet (MW) of the Hydrological Open Air Laboratory in (panel a) June 2006 and (panel b) October–November 2006.

Two time periods were selected for the analysis because of the differences in the record lengths between the main outlet and the tributaries. While the streamflow fluctuations at the main outlet were analyzed for the period 2002–2015, the fluctuations at the tributaries were analyzed for the period 2013–2015. Typical examples of streamflow fluctuations at the main outlet (MW) during a low flow period in summer and in autumn 2006 are presented in Figure 3. The relatively long rainless period during June 2006 resulted in a recession of the streamflow, gradually decreasing from 5 to 3 L/s over 21 days. Due to evapotranspiration, diurnal streamflow fluctuations were superimposed on the recession curve (Figure 3a). In the middle of October 2006, the amplitude of the diurnal streamflow fluctuations was around 0.4 L/s. Following a storm event (28–31 October), the air temperature dropped below freezing point and the diurnal streamflow fluctuations stopped. Even though later the air temperature increased above freezing point, the diurnal streamflow fluctuations did not resume during the rest of the year (Figure 3b).

Nineteen piezometers located in the riparian forest close to the stream have monitored the groundwater level at a resolution of 5 min since 2013. Consistent with the streamflow records, groundwater level fluctuations were analyzed for the period 2013–2015. Five piezometers at different distances from the stream were selected for the analyses. The average depth to the groundwater table between 2013 and 2015 was larger further away from the stream: 0.3 m at BP02 (0.3 m from the stream), 0.4 m at BP07 and H04 (1.6 and 1.4 m from the stream respectively), 2.7 m at H02 (7.4 m from the stream), and 4.3 m at H01 (14.7 m from the stream).

Rainfall has been measured with high temporal resolution (1 min) since 2002 by a weighing rain gauge situated 200 m from the catchment outlet. In 2012 four additional weighing rain gauges were installed in the HOAL (Figure 1).

Since 1986 air temperature has been recorded at 7, 14, and 19 hr by a thermometer and cumulative daily solar radiation has been measured with a pyranometer located about 500 m from the catchment outlet. Since October 2012 air temperature, incoming and outgoing solar and long wave radiation have been measured at the HOAL weather station at 1‐min temporal resolution. Three eddy covariance stations have measured crop evapotranspiration in the HOAL since August 2012.

Streamflow, groundwater level, rainfall, and solar radiation data were aggregated to hourly values for the analysis. Test simulations indicated that the difference between hourly and shorter time steps was negligible during low flows. For the time period 2002–2012, hourly solar radiation on a horizontal surface was estimated from measured cumulative daily solar radiation as a function of the day of the year, the solar time and the latitude (Duffie & Beckman, 2013).

## 3 Methodology

### 3.1 Identification of Periods With Streamflow Fluctuations

In order to analyze the diurnal streamflow fluctuations, periods during streamflow recession with diel streamflow and groundwater level signals were identified for the main outlet, for all tributaries and for five piezometers based on four criteria:

- the day and the night preceding the day had no precipitation,
- the difference between the daily minimum and maximum streamflow or groundwater level exceeded a threshold (see supporting information Table S1),
- the daily minimum streamflow or groundwater level was observed between 8 a.m. and 7 p.m., and
- the daily maximum streamflow or groundwater level was observed either before 3 p.m. or after 9 p.m.

For the selected episodes, the amplitude was estimated as the difference between the daily minimum and maximum streamflow or groundwater level (see supporting information Text S1). If an episode was identified at the main outlet, the streamflow records of the tributaries and the groundwater levels of the piezometers were checked with regard to the presence of fluctuations. If one or more of the criteria above were not matched at the tributaries or at the piezometers, the episode was considered to have zero amplitude; otherwise, the amplitudes were evaluated in a similar way as for the main outlet. The mean streamflow of the episodes was calculated for the same time periods for all streamflow gauges and became 0 only if a tributary dried out.

### 3.2 Modeling of Streamflow Fluctuations

The study proceeded along two approaches of analyzing streamflow fluctuations, that is, direct analyses and model simulations. The spatiotemporal patterns and the seasonal variability of the low flow fluctuations were described through direct analyses of the observations. The model simulations aimed at estimating the time lags of the streamflow response relative to its forcing. A new modeling approach based on a simple impulse response model was used assuming that

- the amplitude of the diurnal streamflow fluctuations is proportional to incoming shortwave solar radiation; that is, solar radiation can be used as a proxy for transpiration (e.g., Renner et al., 2016),
- the temporal pattern of the diurnal streamflow fluctuations can be modeled by an exponential response function to solar radiation, and
- the main recession trend during the low flow period is exponential.

The model has three free parameters (*f*, *λ*, and *α*). The parameter *f* expresses the proportion of the maximum available energy in the entire catchment or subcatchment, which influences the amplitude of the diurnal streamflow fluctuations, assuming that the energy consumed by evapotranspiration is equal to incoming shortwave solar radiation. If this assumption is true, *f* is equal to 1, if the entire maximum catchment energy contributes to diurnal streamflow variations. The parameter *λ* represents the time lag between incoming shortwave solar radiation and the diurnal streamflow fluctuations. The parameter *α* is the recession time scale. If *α* is significantly larger than *λ*, that is, there is a magnitude difference between the two time scales, then a separation of scales exists in the time domain.

Based on assumption (i) evapotranspiration *ET* is estimated from the incoming shortwave solar radiation according to (1)

(1) $$\mathrm{ET}=f\cdot S\cdot \frac{G}{\rho {\Delta H}_{\mathrm{vap}}}$$

where *ET* is evapotranspiration in (L^3^/*T*), *f* is the dimensionless amplitude factor, *S* (L^2^) is the drainage area of the entire catchment or subcatchment, *G* (M/T^3^) is incoming shortwave solar radiation, *ρ* (M/L^3^) is the density of water, and Δ*H*_vap_ (L^2^/*T* ^2^) is the latent heat of vaporization of water. This approach assumes no plant regulation of evapotranspiration. Based on assumption (ii), the evapotranspiration pattern is convoluted with an exponential response function to obtain the evapotranspiration signal in the hydrograph *Q*_*d*_ (L^3^/*T*) according to (2):

(2) $$Q_{d}\left(t\right)=\int_{0}^{t}\mathrm{ET}\left(\tau \right)u\left(t-\tau \right)d\tau$$

where *τ* (*T*) is the integration variable and *u*_*t*_ (1/*T* ^1^) is the response function according to (3):

(3) $$u_{t}=\frac{1}{\lambda }e^{-\frac{t}{\lambda }}$$

where *λ* (*T*) is the time lag and *t* (*T*) is time. Based on assumption (iii), the recession curve *Q*_*r*_ (L^3^/*T*) is expressed as an exponential function (4):

(4) $$Q_{r}=Q_{0}e^{-\frac{t}{\alpha }}$$

where *α* (*T*) is the recession time scale and *Q*_0_ (L^3^/*T*) is set to the maximum measured discharge during the first day of the time period analyzed. Streamflow *Q* (L^3^/*T*) is calculated according to (5), subtracting the evapotranspiration signal from the recession curve:

(5) $$Q=Q_{r}-Q_{d}$$

It is important to note that the model is not a predictive model. It was fitted to the streamflow data using a multiple objective calibration approach (combining the root‐mean‐square error, the amplitude error, and the error of timing, see supporting information Text S2, Chu et al. (2011)) for the purpose of interpreting the streamflow fluctuations and low flow recessions at different times of the year. As the model was fitted to each recession period independently, the result was one parameter set (*f*, *λ*, and *α*) for each episode.

The modeled evapotranspiration volumes were compared with upscaled, literature‐based riparian evapotranspiration volumes (see supporting information Text S3 and Table S2), (Beeson, 2011; Hinckley et al., 1994; Köcher et al., 2008). The groundwater evapotranspiration, that is, the evapotranspiration calculated from the diurnal fluctuations of the shallow groundwater levels, was estimated by the White method (White, 1932) and the empirical method of Gribovszki et al. (2008; see supporting information Text S4).

## 4 Results

### 4.1 Amplitude of the Observed Diurnal Signals

The number of episodes with diurnal variations at the main outlet was 549 and 138 for the two time periods (Table 2). Depending on the runoff generation mechanism, the streamflow data of some tributaries featured diel signals simultaneously with the catchment outlet (approximately 60% of the episodes for the wetlands), while other tributaries were dry during long rainless periods (for instance the erosion gullies E1 and E2). While all periods were used for the direct analyses, periods shorter than 3 days were excluded from the model simulations (Table 2). The mean lengths of the episodes for the direct analyses and model simulations were about 60 and 110 hr, respectively.

**Table 2:** Number of Episodes and Days Included in the Direct Analyses and the Model Simulations and Mean Episode Lengths

Time period	Gauges	Direct analyses			Model simulations		
		Number of episodes	Total number of days in episodes	Mean episode length (hr)	Number of episodes	Total number of days in episodes	Mean episode length (hr)
2002–2015	MW	549	1364	57	185	832	108
2013–2015	MW and tributaries	138	344	56	42	197	113

*Note*. Episodes shorter than 3 days were excluded from the model simulations.

**Figure 4:**
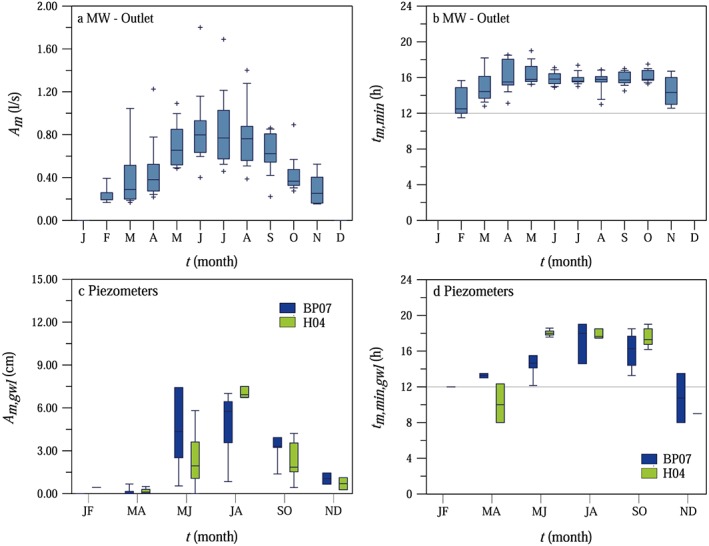
Seasonal variability of the monthly average measured amplitudes *A*_*m*_ of the streamflow diel signals (panel a) and the time of the day when the minimum measured discharge occurs *t*_*m,min*_ (panel b) at MW catchment outlet based on observations during 2002–2015; seasonal variability of the monthly average measured amplitudes *A*_*m,gwl*_ of the groundwater level diel signals (panel c), and the time of the day when the minimum measured groundwater level occurs *t*_*m,min,gwl*_ (panel d) at BP07 and H04 piezometers based on observations during 2013–2015. For the piezometers the results have been lumped into bimonthly bins because of the shorter observation period. Direct analyses, see supporting information Text S1.

The monthly average amplitudes of the streamflow diel signals at the MW catchment outlet and diurnal fluctuations of the groundwater levels at two piezometers (1.6 m from the stream at BP07 and 1.4 m away from the stream at H04) show a clear seasonal pattern (Figures 4a and 4c). The amplitudes of the diurnal streamflow and groundwater level fluctuations increased from spring to summer. During the summer season (May to September) the amplitude of the streamflow fluctuations was usually larger than 0.4 L/s at MW outlet, while during the winter season (November to March) the amplitudes approached 0. The amplitude is controlled by both the energy input, which peaks in the summer, and the efficiency of the plants as they allocate the available energy to transpiration. The very small values of the amplitudes in the late autumn and winter months suggest that the efficiency is the lowest in the winter season. This is consistent with observations (e.g., Figure 3b) that the first late autumn frost terminates the diurnal streamflow fluctuations.

**Figure 5:**
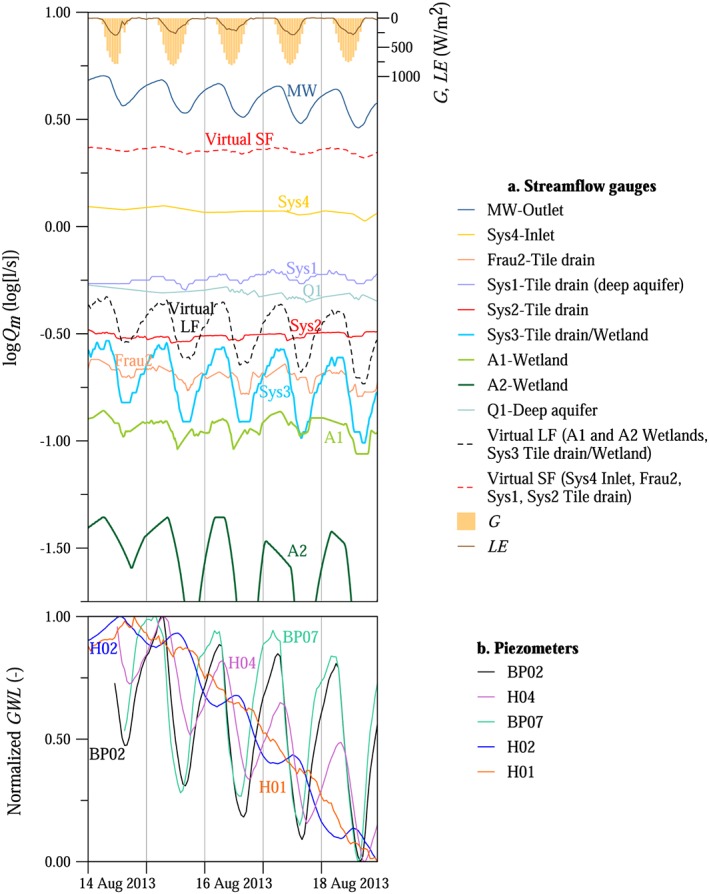
A 5‐day episode in the Hydrological Open Air Laboratory, 14–18 August 2013. (panel a) Measured runoff is shown as solid lines, two virtual gauges (i.e., combination of gauges, LF with large amplitudes and SF with small amplitudes) are shown as dotted lines. Incoming shortwave radiation *G* and latent heat of vaporization *LE* at the weather station are shown at the top. (panel b) Normalized (between 0 and 1) groundwater levels of five piezometers (BP02, H04, BP07, H02, and H01, Figure 1).

**Figure 6:**
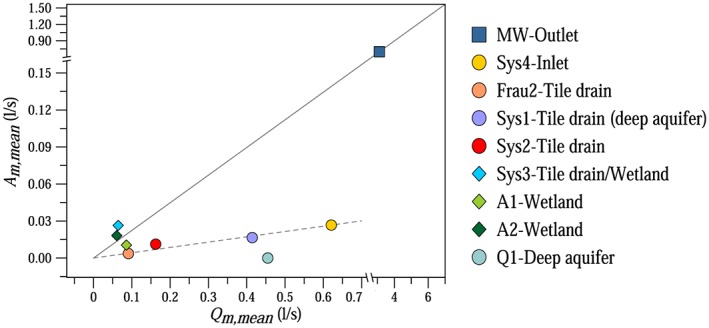
Mean measured streamflow amplitudes (*A*_*m,mean*_) as a function of the mean measured streamflow (*Q*_*m,mean*_) of the episodes at nine stream gauges. Solid line indicates the relative low flow amplitudes of the MW outlet; dashed line indicates the trend of the relative average low flow amplitudes of the tributaries.

The magnitudes of the streamflow fluctuations varied between the locations (Figures 5 and 6). During a 5‐day dry period in August 2013 diel signals were observed for most of the tributaries and piezometers in the HOAL, although with very different magnitudes (Figure 5). The differences between the tributaries are highlighted in Figure 6, which shows the relative amplitudes, that is, the mean measured amplitudes as a function of the mean streamflow of the episodes at the nine gauges of the catchment that usually have nonzero runoff during rainless periods. The other three gauges, the ephemeral Frau1 tile drain and the erosion gullies (E1 and E2), are not included in Figure 6 because they are always dry during rainless periods.

In order to understand from which tributaries the fluctuations detected at MW catchment outlet originate, vegetation cover, dominant soil types, depths to groundwater level, and runoff generation mechanisms were explored for the respective subcatchments. Sys3 tile drain/wetland is located on the left side of the stream, where the depth to groundwater level is small. The drainage area of Sys3 tile drain/wetland is dominated by Kolluvisol around the outlet; the soil texture is characterized by a large percentage of silty loam causing low permeability and generally wet soil conditions. The drainage area around the outlet is covered by forest; the dominant tree types are ash, poplar, and black alder. The root system of these tree species in moisture retaining and organic rich soil types can reach depths of 2 m (Crow, 2005); therefore, they are apparently exceptionally well connected to the shallow groundwater table. Similarly to Sys3 tile drain/wetland, A1 and A2 wetlands were characterized by diel fluctuations with large magnitudes (Figures 5a and 6). The wetlands are also located on the left side of the stream, and due to their high wetness conditions and large riparian forest cover, the trees can be expected to be well connected to the shallow groundwater table as well.

The relative amplitude was 1 magnitude smaller at Sys4 inlet and Sys1, Sys2, Frau2 tile drains compared to the wetlands (Figures 5a and 6). One of the reasons for the smaller relative amplitudes is the different vegetation cover (Table 1). The catchment area of Sys4 inlet and Frau2 tile drains is covered mainly by crop; the forest cover is minimal (Table 1). The fluctuations at these tributaries are possibly caused by the narrow riparian forest zone close to the outlets. Furthermore, Frau2 tile drain enters the main stream in an oblique way from the crop fields so is more exposed to riparian vegetation, unlike Frau1 tile drain that enters perpendicularly and is dry in rainless periods. Even though Sys1 tile drain behaves as a spring according to chemical analyses and it has basically constant contribution to the outflow at the catchment outlet even in the driest months, the root zone of the riparian forest may reach the aquifer, causing diurnal fluctuations of the streamflow. At Sys1 tile drain (deep aquifer) the relative amplitudes of the fluctuations induced by the riparian vegetation were smaller than the relative amplitudes at the wetlands and they were superimposed on the high baseflow rates. The deep aquifer spring Q1, which produces runoff throughout the year from a fractured siltstone layer, is not exposed to the local effect of riparian vegetation; hence, diurnal variations of the outflow were never observed (Figures 5a and 6).

Based on the magnitudes of the observed relative amplitudes and the process reasoning above, the tributaries in the HOAL were separated into two distinct groups, which were lumped into two virtual gauges by taking the sum of the measured streamflow rates. Virtual gauge LF consists of three gauges (A1 and A2 wetlands, and Sys3 tile drain/wetland) with large amplitudes relative to the streamflow rates. Virtual gauge SF consists of four gauges (Sys4 inlet pipe, Frau2, Sys1, and Sys2 tile drainage systems) with small or no diurnal fluctuations in streamflow (Figure 5a).

MW catchment outlet integrates the characteristics of the different runoff mechanisms, and therefore, it can be regarded as a mixture of the different systems. However, Figure 6 suggests that the relative fluctuations at MW (about one fifth of the average low flow, solid line) were much larger than the average of all the tributaries (dashed line). This means that there must be significant additional mechanisms that give rise to the observed fluctuations at MW. About 40% of the streamflow observed at MW enters the stream laterally in a diffuse way (Table 1), mostly through the subsurface, and is not captured by the tributary gauges, which suggests that the remaining part of the fluctuations is related to these diffusive inflows. These are the areas of significant riparian vegetation.

For comparison, Figure 5b shows the groundwater levels normalized to the minimum and maximum levels during the episode for five piezometers (supporting information Table S3), one of them located on the left bank, 1.6 m from the stream (BP07), and four of them on the right bank (BP02, H04, H02, and H01, which are installed 0.3, 1.4, 7.4, and 14.7 m from the stream, respectively). The mean amplitude of the measured groundwater level fluctuations during the 5‐day recession period in August 2013 (Figure 5) was 16.9 cm at BP07 piezometer, while no diurnal fluctuations were observed further away from the stream at H01 piezometer. The amplitude of the groundwater table fluctuations was 13 times larger at BP02 piezometer (0.3 m from the stream) than the water level fluctuations at MW Outlet.

**Figure 7:**
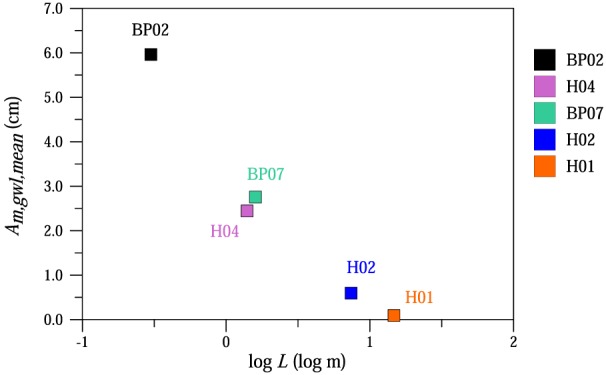
Mean amplitude of the measured groundwater level fluctuations (*A*_*m,gwl,mean*_) as a function of logarithmic distance (log *L*) from the stream.

The impact of distance from the stream on the magnitude of the diurnal fluctuations was further investigated (Figure 7). Similarly to Figure 5b, the mean amplitude of the measured groundwater level fluctuations through the episodes decreased with larger distance from the stream. While the mean amplitude was 6.0 cm at BP02 piezometer 0.3 m from the stream, at H01 piezometer (14.7 m from the stream) it was 0.1 cm. These results indicate that the signal that is propagated into the streamflow decreased with larger distances from the stream.

### 4.2 Timing of the Observed Diurnal Signals

The timing of the diurnal fluctuations, that is, the time of the day when the minimum streamflow or groundwater level occurs, showed a clear seasonal pattern (Figures 4b and 4d). In February and March the daily minimum discharge and groundwater level occurred in the early afternoon. As the season progressed, the daily minimum discharge and groundwater level occurred later in the afternoon, at around 16:00 at MW outlet and around 18:00 at the piezometers. These changes may be related to the response times of the system to the energy input. This phenomenon was analyzed in detail later by the model (section 4.5).

The timing of streamflow fluctuations was compared with the timing of latent heat of vaporization measured by the eddy covariance technique at the weather station (Figure 5a at the top) and groundwater level fluctuations (Figure 5b). The latent heat of vaporization representative of the crop fields and grassland surrounding the weather station lagged behind the incoming solar radiation by 0.5–2 hr (Figure 5a at the top). The phase shift between streamflow and groundwater table fluctuations varied between locations. Piezometers were either in phase with MW, such as closest to the stream at BP02 piezometer (0.3 m away), while further away they changed earlier (BP07) or later (H02 and H04) than MW.

### 4.3 Model Performance

**Figure 8:**
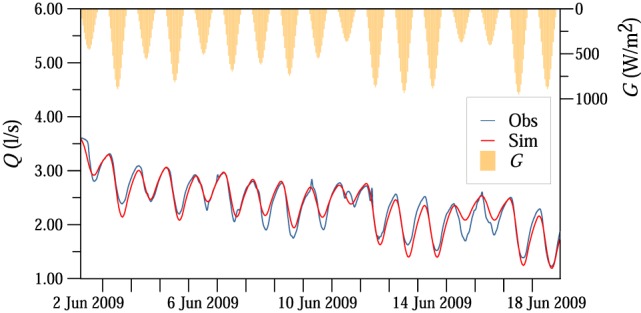
Observed (blue line) and simulated (red line) streamflow fluctuations *Q* at the MW catchment outlet in the period 2–18 June 2009. Incoming shortwave radiation *G* is shown at the top.

**Figure 9:**
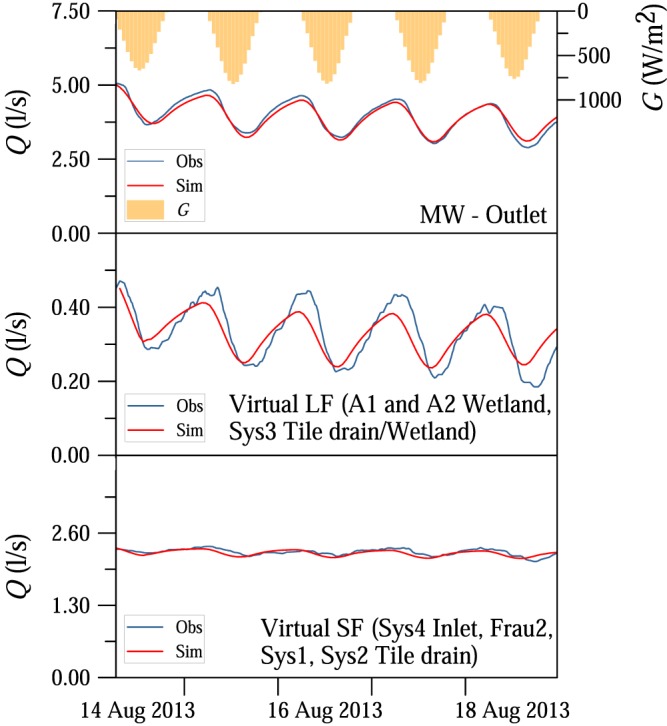
Observed (blue line) and simulated (red line) streamflow *Q* for 14–18 August 2013. The tributaries are lumped into virtual gauge LF (A1 and A2 wetlands, Sys3 tile drain/wetland) and virtual gauge SF (Sys4 inlet, Frau2, Sys1, and Sys2 tile drainage systems). Incoming shortwave radiation *G* is shown at the top.

For the 185 recession periods between 2002 and 2015 (Table 2), the median of the model efficiency, that is, Nash‐Sutcliffe coefficient, for MW was 0.89; the 25th and 75th percentiles were 0.68 and 0.95, respectively (see supporting information Table S4). For autumn, the model efficiency was lower due to the smaller amplitudes and signal‐to‐noise ratios. Figures 8 and 9 show examples of the model fit. A 17‐day recession period in June 2009 had several overcast days (Figure 8). The significant differences in the amplitudes between the days were captured very well by the model. For example, 11 June was an overcast day with a diurnal amplitude of streamflow at MW of only 0.3 L/s compared to the other days with amplitudes of about 1.1 L/s. During a 5‐day period in August 2013 (Figure 9), diel signals were observed at most of the tributaries. Virtual gauge LF (A1 and A2 wetlands and Sys3 tile drain/wetland) was characterized by large amplitudes, while amplitudes at virtual gauge SF (Sys4 inlet, Frau2, Sys1, and Sys2 tile drainage systems) were small.

### 4.4 Process Controls on Amplitude of Diurnal Streamflow Variation

**Figure 10:**
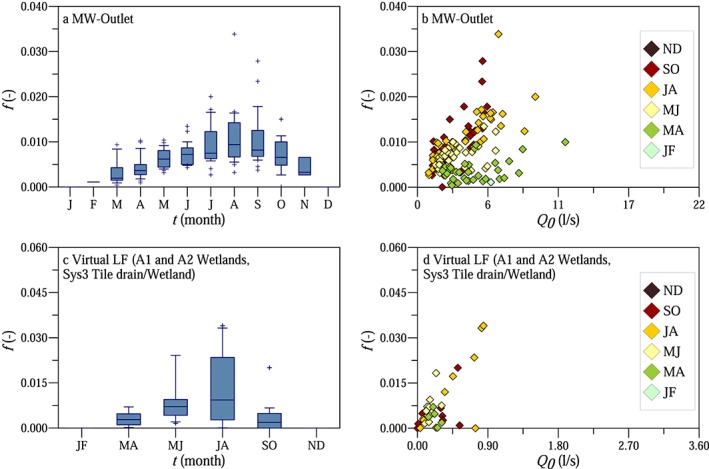
Seasonal evolution of the calibrated amplitude factor *f* (panels a, c) and *f* as a function of the maximum measured discharge *Q*_0_ on the first day of each recession period (panels b, d) for MW catchment outlet (panels a, b) and for Virtual gauge LF comprising the three gauges (A1 and A2 wetlands, Sys3 tile drain/wetland) with large amplitudes (panels c, d). For LF the results have been lumped into bimonthly bins because of the small sample size.

In order to describe the dynamics of the diurnal low flow fluctuations, we analyzed the seasonal evolution of the calibrated model parameters. Figures 10a and 10c show the seasonal variability of the amplitude factor *f* at MW catchment outlet (a) and at Virtual gauge LF (c). Amplitude factor *f* expresses the proportion of maximum available energy in the catchment which affects the diurnal streamflow variations, if the energy consumed by evapotranspiration is equal to incoming shortwave solar radiation. The amplitude factor increased from early spring until the beginning of summer. Assuming that all the entire incoming shortwave radiation is allocated to transpiration, *f* = 0.004 in April and 0.008 in the summer would imply that only a small portion of the available energy in the catchment (0.004 and 0.008, respectively) contributes to the component of transpiration that causes the diurnal streamflow fluctuations.

The seasonal variability of the amplitude factor *f* at virtual gauge LF (A1 and A2 wetlands and Sys3 tile drain/wetland) was similar to the value for the MW catchment outlet (Figure 10c). It increased from early spring (median of 0.003) until the summer (median of 0.009) and decreased in the autumn (median of 0.002). At virtual gauge LF the mean of the amplitude factor *f* was 0.0081, 5 times larger than at virtual gauge SF (0.0018), where the relative amplitude of the fluctuations was 1 magnitude smaller.

Figures 10b and 10d show the calibrated amplitude factor *f* as a function of the maximum measured discharge *Q*_*0*_ on the first day of each recession period. Amplitude factor *f* increased with discharge both for MW catchment outlet (Figure 10b) and even more clearly for virtual gauge LF (Figure 10d).

**Figure 11:**
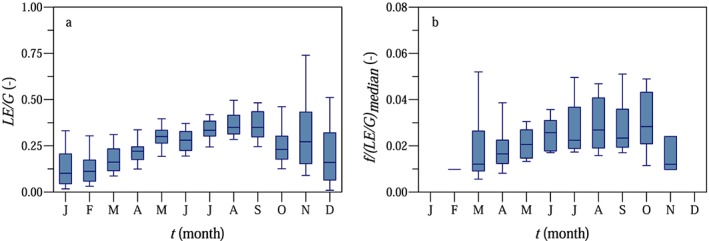
Seasonal variability of the ratio of daily latent heat *LE* measured by eddy covariance and daily incoming shortwave radiation *G* (panel a) and the ratio of the calibrated model parameter *f* and the median of the *LE*/*G* ratios (panel b).

The amplitude factor of the model comprises several factors influencing the diurnal streamflow fluctuations. One of these factors is the efficiency of the vegetation in root water uptake, which we estimated using eddy covariance measurements. Although the flux footprints of the eddy covariance stations located in the crop fields do not cover the riparian zone close to the stream, we estimated the proportion of the incoming solar radiation, which is allocated for transpiration in the crop fields under the climatic conditions in the HOAL. The seasonal variability of the ratio of daily evapotranspiration (expressed as latent heat) and incoming shortwave radiation is shown in Figure 11a. Analyzing only the clear‐sky days between June and August (2012–2014), the average of the incoming shortwave radiation was approximately 25.3 MJ/m^2^/day (or 293 W/m^2^, 10 mm/day), and the latent heat measured in the crop fields was around 7.4 MJ/m^2^/d (or 86 W/m^2^, 3 mm/day). The *LE*/*G* ratio, that is, the plants' efficiency of allocating energy to transpiration, increased from 0.1 in January to about 0.3 in summer (Figure 11a). This means that in summer only about 30% of the incoming shortwave radiation was balanced by latent heat in the crop fields. Assuming similarity between tree and crop transpiration, the ratio of the calibrated model parameter *f* and the median of the *LE*/*G* ratios represents a measure of how well the trees are connected to the stream (Figure 11b). Amplitude factor *f* is essentially the ratio of evapotranspiration seen by the stream and *G*, so *f*/(*LE*/*G*) is the ratio of evapotranspiration seen by the stream and the evapotranspiration measured by the eddy covariance method and hence a measure of the proportion of the available energy in the entire catchment influencing the streamflow fluctuations at the diurnal time scale. Figure 11b suggests that this proportion slightly increased from spring to summer, but the change was much smaller than that of the transpiration efficiency.

### 4.5 Process Controls on Lag Times of Diurnal Streamflow Variation

**Figure 12:**
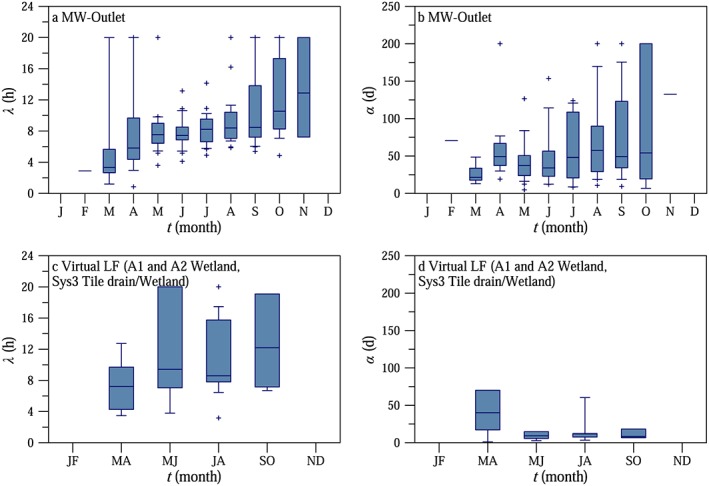
Seasonal evolution of the calibrated model parameter time lag *λ* (panels a, c) and *α* (panels b, d) for MW catchment outlet (panels a, b) and for Virtual gauge LF comprising the three gauges (A1 and A2 wetlands, Sys3 tile drain/wetland) with large amplitudes (panels c, d). For LF the results have been lumped into bimonthly bins because of the small sample size.

The calibrated values of time lag *λ* at MW catchment outlet and virtual gauge LF including only those periods when the model accurately reproduced the observed time series (Nash‐Sutcliffe coefficient > 0.2; 90% and 69% of the modeled episodes were considered for MW outlet and virtual gauge LF, respectively) gradually increased through the year (Figure 12). In March the median of the time lag *λ* was around 3 hr; it increased to 8 hr in May and 11 hr in October. For virtual gauge LF (A1 and A2 wetlands and Sys3 tile drain/wetland) there was a similar increase (Figure 12c). The mean value of the time lag *λ* was higher at Virtual gauge SF (11.1 hr) than at virtual gauge LF (10.3 hr; Table 3).

**Table 3:** Mean of the Three Calibrated Model Parameters for MW Catchment Outlet and the Virtual Gauges in the HOAL

Gauge	Time period	*f* (−)	*λ* (hr)	*α* (days)
MW Catchment Outlet	2013–2015	0.0096	11.3	69.3
MW Catchment Outlet	2002–2015	0.0076	8.9	58.4
Virtual LF (A1 and A2 Wetland, Sys3 Tile drain/Wetland)	2013–2015	0.0081	10.3	19.8
Virtual SF (Sys4 Inlet, Frau2, Sys1, Sys2 Tile drain)	2013–2015	0.0018	11.1	77.6

*Note*. Location of the gauges is shown in Figure 1. HOAL = Hydrological Open Air Laboratory.

The second lag time of the model is the recession time scale *α*. It operates at longer time scales than *λ* and is related to the catchment drainage and evapotranspiration as the catchment dries out during the recession periods. Figures 12b and 12d show the seasonal evolution of the calibrated values of recession time scale *α* at MW catchment outlet (b) and virtual gauge LF (d) for only those periods when the Nash‐Sutcliffe coefficient was larger than 0.2 and there was a clear recession in the hydrographs at MW catchment outlet (66% and 38% of the modeled episodes were considered for MW outlet and virtual gauge LF, respectively). A few episodes had increasing baseflow, which would imply a rainfall input not considered. Therefore, these episodes were not considered in estimating *α*. There was a tendency for time scale *α* to gradually increase from spring to autumn, from a median value of 21 days in March to 54 days in October. According to Table 3, the mean recession time scale *α* was 4 times larger at virtual gauge SF than at virtual gauge LF, where the systems are fed by deeper subsurface flow.

## 5 Discussion

### 5.1 Spatiotemporal Patterns of Streamflow Fluctuations

Several studies analyzed the spatial and temporal differences of diurnal streamflow fluctuations between catchments and in single or nested catchments but focused only on spatially uniform runoff generation mechanisms. For instance, Lundquist and Cayan (2002) analyzed diurnal variations in 100 rivers in the western United States and found a weak correlation between the amplitude of the diurnal cycle and mean monthly temperature, discharge, basin area, and mean basin elevation. They concluded that each catchment was unique and that the spatiotemporal patterns of diel streamflow signals were determined by local physiographic and hydrologic characteristics.

The observation and modeling results presented in this study show that the spatial differences in runoff generation greatly influence the magnitude of the diurnal streamflow fluctuations. We found that the amplitude of the diel streamflow signal relative to the streamflow at the wetlands was 1 magnitude larger than those of the tile drains and the deep aquifers (Figures 5a and 6). The wetlands are the areas in the catchment with high wetness conditions, shallow groundwater table, and large riparian forest cover (Table 1). In contrast, the inlet pipe, the tile drainage systems, and deep aquifers are fed by deeper water sources, covered mainly by crop fields and the depth to the groundwater level is larger than in the wetlands.

The modeling results also reflected the differences in the relative fluctuations between the tributaries. The amplitude factor *f* of the solar radiation‐driven model was larger in the wetlands; that is, a larger proportion of the maximum catchment energy was allocated for transpiration, which influenced the diurnal streamflow fluctuations. The recession time scale *α* of the model was longer for systems fed by deeper subsurface flow (tile drainage systems and deep aquifers) than for the shallower systems as would be expected.

### 5.2 Simplified Process Representation

In this study incoming shortwave radiation proved to be a useful proxy for representing streamflow fluctuations due to riparian transpiration (see section 4.2). Dominant controls on transpiration, that is, stomatal (vapor pressure deficit and conductances) versus boundary layer (radiation) control, depend on the vegetation type, scale, and meteorological conditions (Jarvis & McNaughton, 1986; Martin et al., 2001). Due to the energy that is advected in the form of vapor pressure deficit, daily latent heat flux can exceed the daily sum of net radiation (e.g., Hall et al., 1998, observed such a phenomenon on a few, dry summer days). Therefore, using net radiation as a driver of the diurnal streamflow fluctuations might introduce a bias on longer time scales. Some studies suggested the atmospheric moisture deficit as the main driver of transpiration (e.g., Granier et al., 2000; Szeftel, 2010) or a combination of both energy and vapor pressure deficit (e.g., different versions of the Penman‐Monteith equation). However, other studies showed that transpiration of various tree species was closely related to solar radiation (e.g., Dragoni et al., 2005; Granier et al., 2000; Kume et al., 2008; Oguntunde & Oguntuaseb, 2007). For example, Pieruschka et al. (2010) suggested that the absorbed solar energy by the leaves influenced the stomatal control of transpiration; therefore, solar radiation was an important control on transpiration. Using 110 FLUXNET eddy covariance sites, Boese et al. (2017) observed a substantial transpiration component, also termed as equilibrium transpiration (Jarvis & McNaughton, 1986), which was independent of stomatal conductance and driven by incoming solar radiation. Phillips et al. (1999) showed that diurnal sap flow in a Panamanian humid forest was more correlated with radiation than with atmospheric moisture deficit and Williams et al. (2004) found that transpiration was not correlated with atmospheric vapor pressure deficit in an olive orchard in Morocco. Renner et al. (2016) argued that vapor pressure deficit and wind speed, two variables widely used in evapotranspiration estimations, only slightly increased the predictability of atmospheric demand in a beech forest in Luxembourg.

In this study the evapotranspiration pattern was convoluted with an exponential response function that resulted in a hydrograph shape with convex rising limbs and concave recessions. Kovar and Bacinova (2015) used similar methods when they simulated the diurnal streamflow fluctuations with the Fourier series model. They applied both a linear and an exponential regression to simulate the depletion process and found a very small difference between the two approaches. Dvorakova et al. (2012, 2014) developed and calibrated a linear storage model to describe the recession process, where the actual evapotranspiration was reproduced by simplified Fourier series or sine curves (Dvorakova et al., 2012, 2014). Similar to our results, these studies also showed that the depletion of the catchment storage during low flow conditions could be captured by an exponential function.

### 5.3 Estimated Evapotranspiration Volumes and Rates

The daily transpiration rates depend on the type of vegetation, structure, age, and leaf area index (Farid et al., 2008; Schaeffer et al., 2000). Numerous studies compared evapotranspiration or transpiration rates of different vegetation cover using eddy covariance or sap flow measurements. For example, Granier et al. (2000) found summer transpiration rates of about 4.5 mm/day in a beech forest in France. Water abstraction of willows next to the stream can reach 5.6 mm/day (Marttila et al., 2017). Crop evapotranspiration, typically, is also on the order of 4 mm/day, depending on crop type (e.g., Delzon & Loustau, 2005).

The volumes of the diurnal streamflow fluctuations may be interpreted in two ways. The first and most common interpretation, as presented, for example, in Gribovszki et al. (2010), is to attribute the entire missing volume in the hydrograph to evapotranspiration. This is the assumption underlying our estimations. In terms of volumes, independently from the *LE*/*G* ratio, by integrating equation (1) over the day and taking the average over the episode, the daily average summer (between June and August) evapotranspiration at MW, LF, and LS was 69, 8, and 8 m^3^/day, respectively (Table 4). The tributary influences are expected to propagate synchronously along the main stream due to the short, approximately 50‐ and 80‐min‐long lag times of celerity and velocity, respectively, between the upstream tributaries and MW outlet (Eder et al., 2014). Therefore, this result means that 53 m^3^/day are not accounted for by the tributaries (LF + LS) so needs to be due to the diffusive subsurface inflow to the stream. In other words about 77% (53/69) of the volumes associated with the streamflow fluctuations are related to (hyporheic) exchange along the riparian zone of the main stream.

**Table 4:** Estimated Average Summer (June, July, and August) Evapotranspiration Volumes

Method	Reference gauge/piezometer	Time period	*ET* (m^3^/day)
Incoming shortwave radiation‐driven model	MW Outlet	2002–2015	55
Incoming shortwave radiation‐driven model	MW Outlet	2013–2015	69
Incoming shortwave radiation‐driven model	Virtual gauge LF (A1 and A2 Wetland, Sys3 Tile drain/Wetland)	2013–2015	8
Incoming shortwave radiation‐driven model	Virtual gauge SF (Sys4 Inlet, Frau2, Sys1, Sys2 Tile drain)	2013–2015	8
Upscaling literature‐based evapotranspiration values for the entire riparian zone	—	—	81

The second interpretation of the diurnal streamflow fluctuations is that subsurface flow is controlled by slight changes in the potential gradients that, in turn, are controlled by the diurnal cycle of evapotranspiration as the suction of the roots changes during the day. If this is the case, the summer evapotranspiration rates causing the diurnal streamflow fluctuations are smaller than the estimated ones. Szilágyi et al. (2008) distinguished between a local and an overall hydraulic gradient driving the water transport in the vadose and saturated zones during recession flow periods using a 2‐D finite element numerical model. Voltz et al. (2013) observed an overall relatively small response of the hydraulic gradients during a summer recession period in a steep headwater catchment in Oregon, where the ratio of the cross‐to down‐valley hydraulic gradient showed the largest diurnal fluctuations in wells closest to the stream. Given the topography, where the roots of riparian trees can easily reach into the groundwater, and groundwater levels in the riparian zones themselves fluctuate, this mechanism is not likely important in the HOAL.

In order to compare the mean daily evapotranspiration rates from the main outlet with evapotranspiration rates estimated from the shallow groundwater level fluctuations (*ET*_*G*_), the estimated evapotranspiration volumes (equation (1)) were divided by the product of the catchment area and the calibrated amplitude factor *f*. The comparison indicates that the daily rates from diurnal streamflow fluctuations from the groundwater levels were 7 and 5 mm/day based on the empirical method of Gribovszki et al. (2008) and the White (1932) method, respectively, which was slightly lower than the simulated rates for MW catchment outlet (9.5 mm/day). These results are consistent with the range (8–11 mm/day) found by Gribovszki et al. (2008) in an alder forest in Hungary. As the depth to the groundwater did not exceed 1 m (2013–2015) in the riparian zone within 0.5‐m distance from the stream in the driest summer months, the root system of the trees (especially the 40–70 year olds and >10‐m species) is likely in direct contact with the saturated zone and evapotranspiration estimated from the diurnal groundwater level fluctuations could be close to groundwater evapotranspiration (Dawson, 1996; Shah et al., 2007; Williams et al., 2006).

Because the estimated evapotranspiration volumes could not be validated against measurements of sap flow and stomatal conductances, we estimated the transpired volumes for the entire riparian forest using aerial photographs, a tree survey and literature‐based transpiration values of different tree species (Table 4). The estimated summer transpiration rates (81 m^3^/day) show a good agreement with the modeling results (e.g., incoming shortwave radiation‐driven model set up for MW Outlet, for the time period 2013–15: 69 m^3^/day). The slightly larger values are expected as the literature‐based estimate was calculated for the entire riparian zone (Table 4), which may not fully contribute to the streamflow fluctuations.

### 5.4 Separation of Scales in Time Implies a Separation of Scales in Space

**Figure 13:**
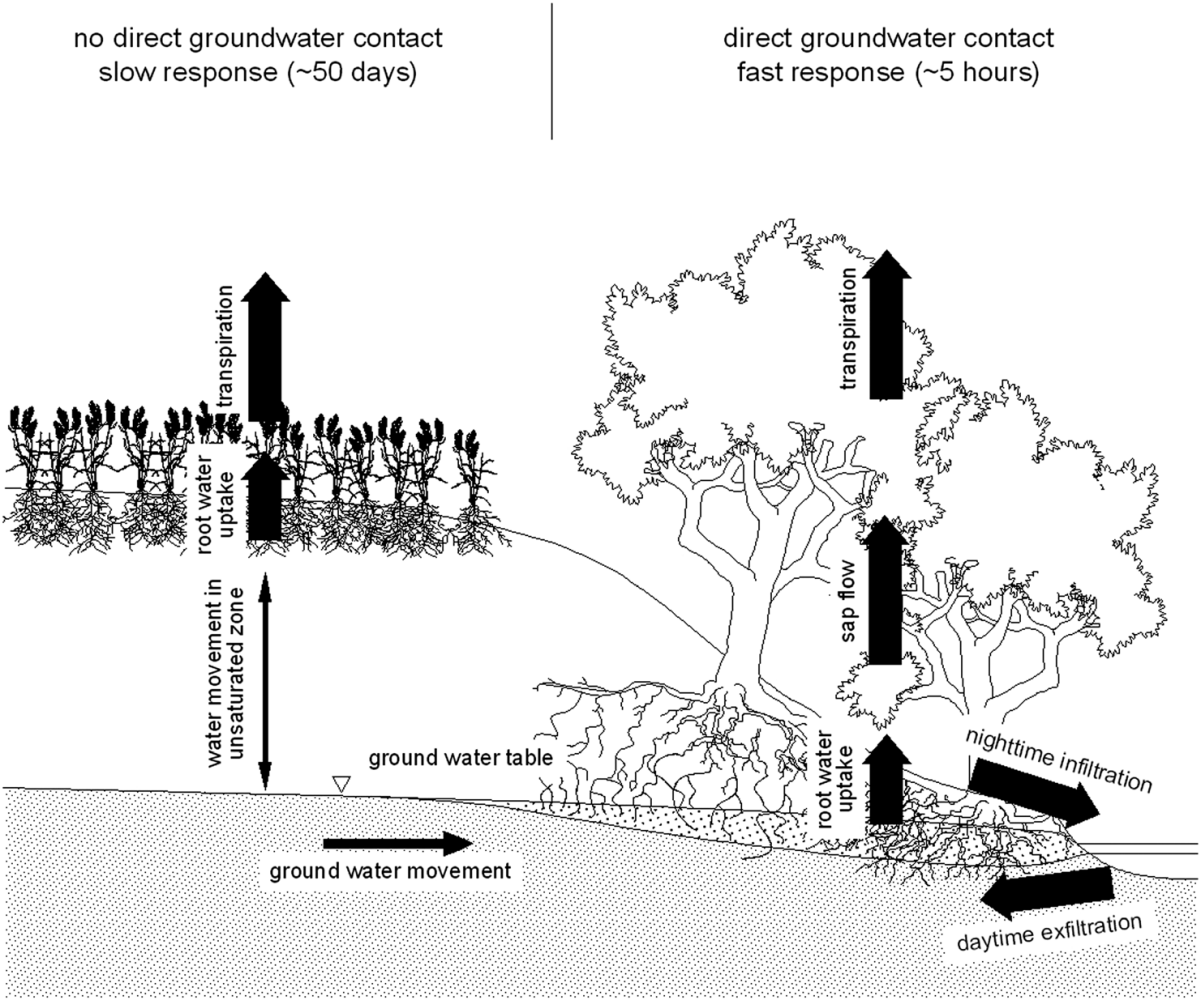
A possible conceptualization of the catchment during low flow periods: schematic of the decoupling of riparian zone fluxes and catchment zone fluxes for the catchment outlet. Width of the arrows indicates the time scale. The thick arrows represent time scales of hours (i.e., well connected at the diurnal scale); the thin arrows represent time scales of weeks or months (i.e., decoupled at the diurnal scale).

The analysis of the streamflow fluctuations during low flow conditions at MW catchment outlet shows that the time lag *λ* between radiation and the diurnal low flow fluctuations gradually increased from 3 to 11 hr as the season progressed. The time lag represents the total response consisting of a cascade of responses, which includes the time lags between radiation and evaporation from the stomata, sap flow in the branches and the stem, root water movement, groundwater movement, and groundwater‐stream interactions (Figure 13). Each component has its own time lag. A number of studies found that the time lag between the diurnal fluctuations of radiation and sap flow in the tree was approximately 30 min for species such as apple trees (Dragoni et al., 2005), beech (Granier et al., 2000), and Japanese cedar (Kumagai et al., 2009). Gartner et al. (2009) found that the sap flow of birch and spruce in the southeastern part of Austria lagged solar radiation by 1 hr during early August. When the soils dried out during a significant drought, the time lags increased to approximately 2.5 hr. Hence, it is likely that the time lag of the mixed vegetation in the riparian zone of the HOAL catchment is also on the order of 0.5 to 2.5 hr. In some of the studies above, the time lag of sap flow from stem to branch was included in the estimates; therefore, this value is also small. Similarly, it is likely that the time lags for root water uptake are small in the riparian zone, where the groundwater table is high, which is typical in the HOAL, especially on the left side of the stream. The remaining time lag components are associated with subsurface processes (groundwater movement and groundwater‐stream interactions). Assuming that the lag components are additive, one would estimate lags of the subsurface processes of about 1.5 hr in early spring to about 9 hr in autumn for MW catchment outlet. The time lags are shorter on those tributaries that are located on the left side of the stream with a western aspect where the groundwater levels are shallow and the riparian forest cover is more dense (virtual gauge LF).

The reason for the longer subsurface lag times in the summer months are presumably a consequence of the soil moisture status of the riparian zone. Along the stream in the HOAL there is probably a mixture of infiltration and exfiltration due to the heterogeneity of the topography in the near‐stream zone. Previous studies (e.g., Caldwell et al., 1998; Voltz et al., 2013) found a reversal of groundwater gradients in the riparian zone due to nighttime infiltration of groundwater into the stream and reversed flow during the day as the trees take up groundwater and part of the stream water to satisfy their transpiration needs (Figure 13). Although we did not observe the complete reversal of the groundwater gradient in the riparian zone with the current measurement setup, a diurnal change in the magnitude of the gradient was observed. The fact that the main part of the time lag is related to the movement of water in the subsurface suggests that the increase in lag times during the summer months is a consequence of the amount of water stored in the near‐stream zone. The subsurface water storage, both soil moisture and groundwater, is largest in spring, which is also indicated by the seasonal maximum in the discharge. Therefore, it is likely that the short lag times in spring are a consequence of the fact that the roots are well connected to the water sources and the celerities are higher. As the season progresses, the catchment gradually dries out and the roots become less connected to the subsurface water storage. When the soils get drier, the unsaturated zone becomes thicker and flow paths get longer and the overall hydraulic conductivity gets smaller. These mechanisms cause an increase in the lag times from 3 to 11 hr for MW catchment outlet. This interpretation is consistent with the findings of Bond et al. (2002), Fonley et al. (2016), Moore et al. (2011), and Wondzell et al. (2007, 2010).

Similarly to time lag *λ*, the recession time scale *α* could be also described by a cascade of responses. This cascade includes the time lags between transpiration of the crop fields in the catchment and root water uptake, water movement in the unsaturated zone, groundwater movement, and groundwater‐stream interactions. While it is difficult to separate the individual components, it is clear that the dynamics of root water uptake and transpiration of crops are faster than the water movement in the unsaturated zone and the groundwater movement. The former operates at a daily scale with significant diurnal variations, while the latter operates at time scales of weeks and months. The differences in the magnitudes of the time scales are indicated in Figure 13 by the thickness of the arrows. Thick arrows represent time scales of hours; thin arrows represent time scales of weeks or months. The recession time scale shows a gradual seasonal increase from 21 to 54 days for MW catchment outlet. This increase is presumably related to the amount of water stored in the subsurface at the catchment scale. As the catchment dries out, groundwater flow seems to follow deeper flow paths, which are associated with longer response times consistent with the findings of Bond et al. (2002). A similar, increasing trend of the recession time scale *α* was found for the subcatchments with shallow groundwater levels (virtual gauge LF) although, overall, the time scales are smaller, as would be expected because of the shorter distances. At subcatchments fed by deeper subsurface flow (virtual gauge SF) the recession time scale *α* was longer indicating a reduced dependence on riparian processes.

Observations and model simulations showed that the diurnal signal observed in streamflow at different outlet points of the HOAL mainly originates from diurnal fluctuations in the riparian evapotranspiration. There is a clear spring onset, a late autumn offset (Figure 3b) of the diurnal diel signal in streamflow and a clear seasonal pattern in the amplitudes and timings (Figure 4). While the first harvest of the crop fields in July does not influence the amplitude of the diurnal signal in the stream (Figure 4), the amplitudes start to decrease only in the autumn months and the first frost when the trees in the riparian zone next to the stream drop their leaves terminates the diurnal streamflow fluctuations. Streamflow from tributaries with large riparian forest cover fluctuated more than streamflow from tributaries which were covered mainly by crop fields and fed by deep aquifers. These observations imply that the riparian zone is the main driver of the diurnal streamflow fluctuations. The results of a solar radiation‐driven model showed that only a small proportion of the maximum available catchment energy induced the diurnal streamflow fluctuations (Figure 10). This proportion was larger during higher baseflow conditions, and it increased from spring to summer and decreased in autumn. The daily minimum streamflow occurred later as the season progressed, which was also reflected in the time lags of the solar radiation‐driven model. The change in the timing of the daily minimum streamflow and the increase in lag time are likely related to the drying of the catchment during the summer and autumn, which may lead to a partial disconnect of the riparian zone and the stream. This is consistent with the findings of previous studies, such as Bond et al. (2002) and Wondzell et al. (2007, 2010) who showed that the area contributing to streamflow fluctuations decreased as the catchment gradually dried out.

A clear separation of scales (Blöschl & Sivapalan, 1995) exists in the time domain, which is apparent in the streamflow signal. Conceptually, time scales and space scales of variability are linked through their characteristic velocities; therefore, a separation of scales in the space domain would be expected, if a separation of scales in the time domain exists (Skøien et al., 2003). There is also interaction across the diurnal and seasonal time scales. The diurnal low flow fluctuations due to riparian and near‐riparian transpiration are modulated by the soil moisture state at the seasonal time scales. Conversely, daily transpiration contributes to the seasonal totals. This interaction across time scales is reminiscent of the effects of climate variability on floods—in a direct way through the seasonal variability of storm characteristics and indirectly through the seasonality of rainfall and evapotranspiration that affect the antecedent catchment conditions for individual storm events (Sivapalan et al., 2005).

The streamflow and piezometer observations imply that at the daily time scale, riparian evapotranspiration induces the diurnal streamflow fluctuations, while most of the catchment evapotranspiration, such as evapotranspiration from the crop fields further away from the stream does not contribute to these fluctuations. This implication was confirmed by piezometer data. We found that the amplitudes of the fluctuations in the riparian zone within 2‐m distance from the stream were significant and did not find significant fluctuations 15 m from the stream. This is consistent with the findings of Reigner (1966), who also found that the amplitudes were significant within 2‐m distance from the stream. This behavior is also apparent in the time domain. As the first frost occurs, the low flow fluctuations at MW stop within days (Figure 3b). A similar behavior has been observed by Goodrich et al. (2000) and for groundwater fluctuations by Lautz (2008). Deutscher et al. (2016) distinguished between two distinct parts of the catchment with different connectivity, and connectivity may also exist in terms of soil moisture patterns (Western et al., 1998). Conversely, there is a quick onset of the fluctuations in spring at the beginning of the growing season, when the vegetation is growing. Preferred states and switching behavior associated with thresholds seem to be more common characteristics than what is usually assumed (Blöschl & Zehe, 2005; Zehe & Sivapalan, 2009). Spatial patterns of hydrologic dynamics may help identify preferred states (Blöschl, 2006; Grayson et al., 1997). Analyzing spatial patterns may also help to address the difficulties in predicting the whole‐catchment water balance from observations at the local scale (Thompson et al., 2011).

## 6 Conclusions

This study investigated the spatial and temporal patterns of diurnal low flow fluctuations for different runoff generation mechanisms in a 66 ha Austrian experimental catchment, the HOAL. Our results showed that:

‐The ratio of streamflow fluctuations and mean streamflow was around 0.3 for wetlands, where the riparian forest cover is the largest and the depth to the groundwater table does not exceed 1 m. The amplitudes were much smaller for tile drainage systems and springs that are fed by deeper subsurface flow, where the dominant land cover is crop and the ratio is around 0.04.

‐The separation of scales in the time domain could be reproduced by a solar radiation‐driven model. Lag times between radiative forcing and evapotranspiration increased from 3 to 11 hr from spring to autumn as the catchment became more disconnected from the stream. The recession time scales increased from 25 days in spring to 60 days in autumn, which was likely a consequence of the decreasing storage of subsurface water at the catchment scale.

‐A separation of scales in the time domain is apparent in the streamflow signal, that is, diurnal and seasonal fluctuations induced by transpiration, implies a separation of scales in the space domain: the diurnal streamflow fluctuation are driven by the riparian zone along the main stream, while most of the catchment (the crop fields located further away from the stream) did not affect the diel signals. This interpretation is supported by the groundwater level data.

## Supporting information



Text S1

Text S2

Text S3

Text S4

Table S1

Table S2

Table S3

Table S4
