# Supplementary material for: Separation of Scales in Transpiration Effects on Low Flows: A Spatial Analysis in the Hydrological Open Air Laboratory
Source: Water Resour Res. 2018 Sep 10;54(9):6168–88. doi: 10.1029/2017WR022037 (PMC6221015; doi:10.1029/2017WR022037)
Supplement: Supplementary file 1 — Text S1 [file WRCR-54-6168-s001.docx]

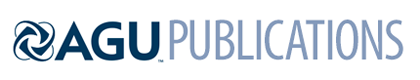


*Water Resources Research*

Supporting Information for

**Separation of scales in transpiration effects on low flows – A spatial analysis in the Hydrological Open Air Laboratory (HOAL)**

B. Széles^1,2^, M. Broer^3^, J. Parajka^1,2^, P. Hogan^1^, A. Eder^1,4^, P. Strauss^4^, and G. Blöschl^1,2^

^1^Centre for Water Resource Systems, Vienna University of Technology, Karlsplatz 13, 1040 Vienna, Austria

^2^Institute of Hydraulic Engineering and Water Resources Management, Vienna University of Technology, Karlsplatz 13/222, 1040 Vienna, Austria

^3^Umweltbundesamt, Environment Agency Austria, Spittelauer Lände 5, 1090 Vienna, Austria

^4^Federal Agency of Water Management, Institute for Land and Water Management Research, Pollnbergstraße 1, 3252 Petzenkirchen, Austria

**Contents of this file**

Text S1

**Introduction**

Text S1 contains details on the calculations applied to quantify the amplitudes of the diurnal fluctuations.

Text S1. Amplitudes of the diurnal fluctuations

A difference was made between the falling and the rising limbs of the diel signals when the amplitudes of the diurnal fluctuations were calculated. For each day in each episode the daily minimum *Q_m,min_* (L^3^T^-1^) and maximum *Q_m,max_* (L^3^T^-1^) measured discharge values were selected. Those measured amplitudes *a_m_* (L^3^T^-1^) which describe the falling limbs of the diurnal fluctuations were calculated according to (S1.1) starting from the first day

| $a_{m}\left( 2j-1 \right)=\left\vert Q_{m,max}\left( j \right)-Q_{m,min}\left( j \right) \right\vert$ | (S1.1) |
| --- | --- |

where *j* denotes the day within an episode.

Measured amplitudes *a_m_* (L^3^T^-1^) describing the rising limbs of the diurnal fluctuations were calculated according to (S1.2) starting from the second day

| $a_{m}\left( 2j-2 \right)=\left\vert Q_{m,max}\left( j \right)-Q_{m,min}\left( j-1 \right) \right\vert$ | (S1.2) |
| --- | --- |

Measured amplitude *A_m_* describing an episode was calculated as the mean of *a_m_*. Simulated amplitudes *a* and the mean value *A* were obtained in a similar way.

The average measured amplitude of the groundwater level fluctuations *A_m,gwl_* was evaluated similarly based on the daily minimum and maximum measured groundwater levels.
