# Supplementary material for: Separation of Scales in Transpiration Effects on Low Flows: A Spatial Analysis in the Hydrological Open Air Laboratory
Source: Water Resour Res. 2018 Sep 10;54(9):6168–88. doi: 10.1029/2017WR022037 (PMC6221015; doi:10.1029/2017WR022037)
Supplement: Supplementary file 2 — Text S2 [file WRCR-54-6168-s002.docx]

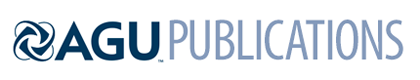


*Water Resources Research*

Supporting Information for

**Separation of scales in transpiration effects on low flows – A spatial analysis in the Hydrological Open Air Laboratory (HOAL)**

B. Széles^1,2^, M. Broer^3^, J. Parajka^1,2^, P. Hogan^1^, A. Eder^1,4^, P. Strauss^4^, and G. Blöschl^1,2^

^1^Centre for Water Resource Systems, Vienna University of Technology, Karlsplatz 13, 1040 Vienna, Austria

^2^Institute of Hydraulic Engineering and Water Resources Management, Vienna University of Technology, Karlsplatz 13/222, 1040 Vienna, Austria

^3^Umweltbundesamt, Environment Agency Austria, Spittelauer Lände 5, 1090 Vienna, Austria

^4^Federal Agency of Water Management, Institute for Land and Water Management Research, Pollnbergstraße 1, 3252 Petzenkirchen, Austria

**Contents of this file**

Text S2

**Introduction**

Text S2 contains information on the optimization procedure.

Text S2. Optimization procedure

Before the optimization procedure, we applied a simple smoothing algorithm on the *Q_m_* observed discharge data according to (S2.1)

| ${Q_{m}}^{'}(t_{i})=\frac{1}{2}\left( 1-w \right)Q_{m}(t_{i-1})+wQ_{m}(t_{i})+\frac{1}{2}\left( 1-w \right)Q_{m}(t_{i+1})$ | (S2.1) |
| --- | --- |

where *w* is 0.5, *i* is the hourly time step.

The multiple objective calibration approach is based on the shuffled complex evolution method with principal components analysis (*Chu et al.*, 2011). The compound objective function incorporates additional information on the timing and amplitudes of the fluctuations, thereby providing a better fit between the measured and simulated streamflow. The model was fitted to each of the recession periods independently by minimizing the objective function *Z* according to (S2.2)

| $Z=w_{1}Z_{E}+w_{2}Z_{A}+w_{3}Z_{T}$ | (S2.2) |
| --- | --- |

where *Z_E_* (L^3^T^-1^) is the root mean square error (S2.3), *Z_A_* (L^3^T^-1^) is the amplitude error (S2.4), *Z_T_* (T) is the error of timing (S2.5). *w_1_*, *w_2_*, *w_3_* weights were assigned in test simulations and sensitivity analyses as *w_1_*=10, *w_2_*=1, *w_3_*=0.1 for MW catchment outlet and *w_1_*=100, *w_2_*=10, *w_3_*=0.1 for the tributaries. The results were only moderately sensitive to the selection of weights. *Z_E_* was calculated as the root of the average of the squared difference between the simulated and observed discharge according to (S2.3)

| $Z_{E}=\sqrt{\frac{\sum_{i=1}^{N} \left( Q\left( t_{i} \right)-{Q_{m}}^{'}\left( t_{i} \right) \right)^{2}}{N}}$ | (S2.3) |
| --- | --- |

where *Q* is the simulated streamflow, *Q_m_’* is the observed and smoothed streamflow, *N* is the number of time steps in one episode. *Z_A_* amplitude error expresses the difference between the *a* (L^3^T^-1^) simulated amplitudes and *a_m_’* (L^3^T^-1^) amplitudes of the measured and smoothed discharge time series according to (S2.4)

| $Z_{A}=\sum_{k=1}^{2M-1} \left\vert a\left( k \right)-{a_{m}}^{'}\left( k \right) \right\vert$ | (S2.4) |
| --- | --- |

where *M* is the number of days within one episode.

*Z_T_* is the error of timing which is defined as the difference between the time of the simulated and measured minimum and maximum discharge within one day according to (S2.5)

| $Z_{T}=\sum_{j=1}^{M} \left\vert t_{max}\left( j \right)-{t_{m,max}}^{'}\left( j \right) \right\vert+\sum_{j=1}^{M} \left\vert t_{min}\left( j \right)-{t_{m,min}}^{'}\left( j \right) \right\vert$ | (S2.5) |
| --- | --- |

where *t_max_* is the time of the maximum simulated discharge within one day, *t_m,max_’* is the time of the maximum observed and smoothed discharge within one day, *t_min_* is the time of the minimum simulated discharge within one day, *t_m,min_’* is the time of the minimum observed and smoothed discharge within one day.

In certain cases (e.g. when the tributaries did not show the diel signals and the procedure weakly converged) the compound objective function (S2.2) was simplified and only the root mean square error according to (S2.3) was applied.
