# Supplementary material for: Separation of Scales in Transpiration Effects on Low Flows: A Spatial Analysis in the Hydrological Open Air Laboratory
Source: Water Resour Res. 2018 Sep 10;54(9):6168–88. doi: 10.1029/2017WR022037 (PMC6221015; doi:10.1029/2017WR022037)
Supplement: Supplementary file 3 — Text S3 [file WRCR-54-6168-s003.docx]

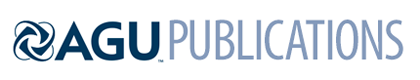


*Water Resources Research*

Supporting Information for

**Separation of scales in transpiration effects on low flows – A spatial analysis in the Hydrological Open Air Laboratory (HOAL)**

B. Széles^1,2^, M. Broer^3^, J. Parajka^1,2^, P. Hogan^1^, A. Eder^1,4^, P. Strauss^4^, and G. Blöschl^1,2^

^1^Centre for Water Resource Systems, Vienna University of Technology, Karlsplatz 13, 1040 Vienna, Austria

^2^Institute of Hydraulic Engineering and Water Resources Management, Vienna University of Technology, Karlsplatz 13/222, 1040 Vienna, Austria

^3^Umweltbundesamt, Environment Agency Austria, Spittelauer Lände 5, 1090 Vienna, Austria

^4^Federal Agency of Water Management, Institute for Land and Water Management Research, Pollnbergstraße 1, 3252 Petzenkirchen, Austria

**Contents of this file**

Text S3

**Introduction**

Text S3 contains information on a literature based evapotranspiration estimation method.

Text S3. Upscaling of literature based riparian evapotranspiration volumes

Based on the heterogeneity of the vegetation, i.e. the dominant tree types, the riparian zone was divided into three parts (upstream area is dominated by field maple and black alder, middle section by poplar, downstream by ash). For each part, a 10x10 m representative area was selected, the dominant tree types were counted and based on tree height and trunk diameter the transpiration rates for the different tree species for the growing season were estimated based on literature values (see Supporting Information Table S2). According to previous studies the dominant trees (one third of the total tree number) account for about two-thirds of the stand level water loss (Čermák et al., 2004), therefore the water loss of the dominant tree types was multiplied by a factor of 1.5 in order to take into account the transpiration of the understory vegetation. The water use of the representative 100 m^2^ patch was upscaled to the entire tree stand. The spatial extent of the tree stand was estimated based on aerial photographs.
