# Supplementary material for: Separation of Scales in Transpiration Effects on Low Flows: A Spatial Analysis in the Hydrological Open Air Laboratory
Source: Water Resour Res. 2018 Sep 10;54(9):6168–88. doi: 10.1029/2017WR022037 (PMC6221015; doi:10.1029/2017WR022037)
Supplement: Supplementary file 4 — Text S4 [file WRCR-54-6168-s004.docx]

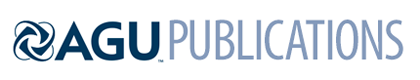


*Water Resources Research*

Supporting Information for

**Separation of scales in transpiration effects on low flows – A spatial analysis in the Hydrological Open Air Laboratory (HOAL)**

B. Széles^1,2^, M. Broer^3^, J. Parajka^1,2^, P. Hogan^1^, A. Eder^1,4^, P. Strauss^4^, and G. Blöschl^1,2^

^1^Centre for Water Resource Systems, Vienna University of Technology, Karlsplatz 13, 1040 Vienna, Austria

^2^Institute of Hydraulic Engineering and Water Resources Management, Vienna University of Technology, Karlsplatz 13/222, 1040 Vienna, Austria

^3^Umweltbundesamt, Environment Agency Austria, Spittelauer Lände 5, 1090 Vienna, Austria

^4^Federal Agency of Water Management, Institute for Land and Water Management Research, Pollnbergstraße 1, 3252 Petzenkirchen, Austria

**Contents of this file**

Text S4

**Introduction**

Text S4 contains information on a literature based evapotranspiration estimation method.

Text S4. Estimation of evapotranspiration using diurnal groundwater level fluctuations

Evapotranspiration was calculated based on the White method (White, 1932) and the empirical method of Gribovszki et al. (2008) using piezometer measurements from the left side of the stream (piezometer BP07). Specific yield was estimated using Table 1 from Loheide et al. (2005). According to a detailed soil survey in the HOAL, the surrounding area of BP07 is dominated by silt (silt: 76.70%, clay: 14.45%, sand: 8.85%), therefore the readily available specific yield was chosen to be S_y_=0.037.
