# Supplementary material for: Separation of Scales in Transpiration Effects on Low Flows: A Spatial Analysis in the Hydrological Open Air Laboratory
Source: Water Resour Res. 2018 Sep 10;54(9):6168–88. doi: 10.1029/2017WR022037 (PMC6221015; doi:10.1029/2017WR022037)
Supplement: Supplementary file 5 — Table S1 [file WRCR-54-6168-s005.docx]

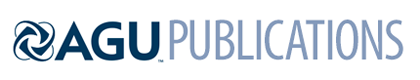


*Water Resources Research*

Supporting Information for

**Separation of scales in transpiration effects on low flows – A spatial analysis in the Hydrological Open Air Laboratory (HOAL)**

B. Széles^1,2^, M. Broer^3^, J. Parajka^1,2^, P. Hogan^1^, A. Eder^1,4^, P. Strauss^4^, and G. Blöschl^1,2^

^1^Centre for Water Resource Systems, Vienna University of Technology, Karlsplatz 13, 1040 Vienna, Austria

^2^Institute of Hydraulic Engineering and Water Resources Management, Vienna University of Technology, Karlsplatz 13/222, 1040 Vienna, Austria

^3^Umweltbundesamt, Environment Agency Austria, Spittelauer Lände 5, 1090 Vienna, Austria

^4^Federal Agency of Water Management, Institute for Land and Water Management Research, Pollnbergstraße 1, 3252 Petzenkirchen, Austria

**Contents of this file**

Table S1

**Introduction**

Table S1 shows the thresholds, i.e. minimum difference between the daily minimum and maximum streamflow rate, for each gauge used for the automatic episode identification.

Table S1. Minimum difference between the daily minimum and maximum streamflow and water level for each gauge, automatic episode identification

| **Gauge** | **Threshold** |
| --- | --- |
| MW - Outlet | 0.1300 l/s |
| Sys4 - Inlet pipe | 0.0700 l/s |
| Frau2 - Tile drain | 0.0006 l/s |
| Sys1 - Tile drain (deep aquifer) | 0.0130 l/s |
| Sys2 - Tile drain | 0.0045 l/s |
| Sys3 - Tile drain/Wetland | 0.0018 l/s |
| A1 - Wetland | 0.0120 l/s |
| A2 - Wetland | 0.0040 l/s |
| Piezometers | 0.5 cm |
