# Supplementary material for: Separation of Scales in Transpiration Effects on Low Flows: A Spatial Analysis in the Hydrological Open Air Laboratory
Source: Water Resour Res. 2018 Sep 10;54(9):6168–88. doi: 10.1029/2017WR022037 (PMC6221015; doi:10.1029/2017WR022037)
Supplement: Supplementary file 6 — Table S2 [file WRCR-54-6168-s006.docx]

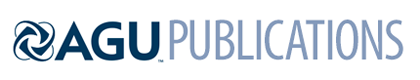


*Water Resources Research*

Supporting Information for

**Separation of scales in transpiration effects on low flows – A spatial analysis in the Hydrological Open Air Laboratory (HOAL)**

B. Széles^1,2^, M. Broer^3^, J. Parajka^1,2^, P. Hogan^1^, A. Eder^1,4^, P. Strauss^4^, and G. Blöschl^1,2^

^1^Centre for Water Resource Systems, Vienna University of Technology, Karlsplatz 13, 1040 Vienna, Austria

^2^Institute of Hydraulic Engineering and Water Resources Management, Vienna University of Technology, Karlsplatz 13/222, 1040 Vienna, Austria

^3^Umweltbundesamt, Environment Agency Austria, Spittelauer Lände 5, 1090 Vienna, Austria

^4^Federal Agency of Water Management, Institute for Land and Water Management Research, Pollnbergstraße 1, 3252 Petzenkirchen, Austria

**Contents of this file**

Table S2

**Introduction**

Table S2 contains information on literature based transpiration values for different tree species.

Table S2. Literature based transpiration values

| **Tree type** | **Reference** | **Diameter (cm)** | **Water use (l/d)** |
| --- | --- | --- | --- |
| Ash | Köcher et al. (2008) | 39.5 | 8.7 |
| Poplar | Hinckley et al. (1994) | 16.0 | 50.0 |
| Field maple | Beeson (2011) | 11.0 | 55.0 |
