# Supplementary material for: Separation of Scales in Transpiration Effects on Low Flows: A Spatial Analysis in the Hydrological Open Air Laboratory
Source: Water Resour Res. 2018 Sep 10;54(9):6168–88. doi: 10.1029/2017WR022037 (PMC6221015; doi:10.1029/2017WR022037)
Supplement: Supplementary file 7 — Table S3 [file WRCR-54-6168-s007.docx]

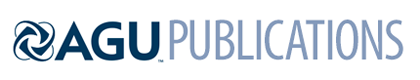


*Water Resources Research*

Supporting Information for

**Separation of scales in transpiration effects on low flows – A spatial analysis in the Hydrological Open Air Laboratory (HOAL)**

B. Széles^1,2^, M. Broer^3^, J. Parajka^1,2^, P. Hogan^1^, A. Eder^1,4^, P. Strauss^4^, and G. Blöschl^1,2^

^1^Centre for Water Resource Systems, Vienna University of Technology, Karlsplatz 13, 1040 Vienna, Austria

^2^Institute of Hydraulic Engineering and Water Resources Management, Vienna University of Technology, Karlsplatz 13/222, 1040 Vienna, Austria

^3^Umweltbundesamt, Environment Agency Austria, Spittelauer Lände 5, 1090 Vienna, Austria

^4^Federal Agency of Water Management, Institute for Land and Water Management Research, Pollnbergstraße 1, 3252 Petzenkirchen, Austria

**Contents of this file**

Table S3

**Introduction**

Table S3 shows the minimum and maximum groundwater level at five piezometers during a five-day period, 14-18 August 2013.

Table S3. Minimum and maximum groundwater level during a five-day episode, 14-18 August 2013

| **Piezometer** | **Minimum *GWL* (m asl)** | **Maximum *GWL* (m asl)** |
| --- | --- | --- |
| BP02 | 258.23 | 258.50 |
| BP07 | 270.87 | 270.97 |
| H01 | 261.70 | 261.79 |
| H02 | 258.04 | 258.61 |
| H04 | 265.07 | 265.25 |
