# Supplementary material for: Separation of Scales in Transpiration Effects on Low Flows: A Spatial Analysis in the Hydrological Open Air Laboratory
Source: Water Resour Res. 2018 Sep 10;54(9):6168–88. doi: 10.1029/2017WR022037 (PMC6221015; doi:10.1029/2017WR022037)
Supplement: Supplementary file 8 — Table S4 [file WRCR-54-6168-s008.docx]

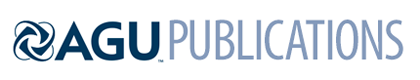


*Water Resources Research*

Supporting Information for

**Separation of scales in transpiration effects on low flows – A spatial analysis in the Hydrological Open Air Laboratory (HOAL)**

B. Széles^1,2^, M. Broer^3^, J. Parajka^1,2^, P. Hogan^1^, A. Eder^1,4^, P. Strauss^4^, and G. Blöschl^1,2^

^1^Centre for Water Resource Systems, Vienna University of Technology, Karlsplatz 13, 1040 Vienna, Austria

^2^Institute of Hydraulic Engineering and Water Resources Management, Vienna University of Technology, Karlsplatz 13/222, 1040 Vienna, Austria

^3^Umweltbundesamt, Environment Agency Austria, Spittelauer Lände 5, 1090 Vienna, Austria

^4^Federal Agency of Water Management, Institute for Land and Water Management Research, Pollnbergstraße 1, 3252 Petzenkirchen, Austria

**Contents of this file**

Table S4

**Introduction**

Table S4 contains information on the model performance statistics.

Table S4. Model performance statistics

| **Nash Sutcliffe Coefficient** | **MW Outlet**  **2002-2015** | **MW Outlet**  **2013-2015** | **Virtual gauge LF (A1 and A2 Wetland, Sys3 Tile drain/Wetland)** | **Virtual gauge SF (Sys4 Inlet, Frau2, Sys1, Sys2 Tile drain)** |
| --- | --- | --- | --- | --- |
| Median | 0.89 | 0.72 | 0.45 | 0.48 |
| 25^th^ percentile | 0.68 | 0.31 | 0.04 | 0.01 |
| 75^th^ percentile | 0.95 | 0.89 | 0.73 | 0.73 |
